# Functional remission in schizophrenia disorder : prevalence and predictors

**DOI:** 10.1192/j.eurpsy.2026.12167

**Published:** 2026-07-31

**Authors:** R. Kammoun, H. Hsine, H. Ghabi, H. Nefzi, M. Karoui, F. Ellouze

**Affiliations:** 1 psychiatry, Razi hospital, Tunis, Tunisia; 2 psychiatry, La metairie Clinic, Nyon, Switzerland

## Abstract

**Introduction:**

Following the development of criteria for symptomatic remission, researchers are now focusing on the social and occupational functioning of individuals with schizophrenia.

**Objectives:**

This study aimed to assess the prevalence of functional remission and to identify its predictors in patients with schizophrenia.

**Methods:**

We conducted a cross-sectional, retrospective and descriptive study in the psychiatry department “G”, in Razi hospital (Tunis), between October 2023 and March 2024. Sixty patients suffering from schizophrenia were included. Functional status was explored with the global assessment of functioning scale (GAF), the social and occupational functioning assessment scale (SOFAS) and the social autonomy scale (SAS). Symptomatic remission was defined according to the remission in schizophrenia working group (RSWG) criteria. All participants were assessed with the positive and negative syndrom Scale (PANSS), the Calgary depression scale in schizophrenia (CDSS), the Birchwood insight scale (BIS), the Simpson and Angus extrapyramidal side effects scale, the mini mental state exam (MMSE), the frontal assessment battery (FAB) and the clock test.

**Results:**

Rates of functional remission were respectively: 63.3% at the GAF scale, 48.3% at the SOFAS and 51.7% at the SAS. Predictors of functional status were: duration of untreated psychosis, schizophrenia subtype, negative symptoms, symptomatic remission, depression and employment status. Executive functions and verbal fluency were also among predictors of functional remission.

**Conclusions:**

Our results confirm the disabling nature of schizophrenia, yet they draw attention to the complexity of the functional remission dimension as well as the possibility of social and professional reintegration with the disease.

**Disclosure of Interest:**

None Declared

